# Cooperation in an Assortative Matching Prisoners Dilemma Experiment with Pro-Social Dummies

**DOI:** 10.1038/s41598-019-50083-6

**Published:** 2019-09-20

**Authors:** Chun-Lei Yang, Ching-Syang Jack Yue

**Affiliations:** 1 Economics Experimental Lab, Nanjing Audit University, 86 Yushanxi Road, Nanjing, 211815 China; 2 Department of Statistics, National Chengchi University, Taipei, Taiwan, ROC

**Keywords:** Evolutionary ecology, Evolutionary theory

## Abstract

Assortative matching (AM) can be theoretically an effective means to facilitate cooperation. We designed a controlled lab experiment with three treatments on multi-round prisoner’s dilemma. With matching based on weighted history (WH) as surrogate for AM, we show that adding pro-social dummies to the WH treatment may significantly improve cooperation, compared to both the random matching and the WH treatment. In society where assortative matching is effective and promoted by the underlying culture, institutional promotion of virtue role models can be interpreted as generating additional pro-social dummies, so as to move the initial state of cooperators into the basin of attraction for a highly cooperative polymorphic equilibrium.

## Introduction

Cooperation in social dilemma games has attracted the attention of many scientific disciplines. Various settings have been investigated that induce significantly higher level of cooperation than the baseline of random matching one-shot encounters without community information. Models of repeated partnership resort to trigger and other strategies (direct reciprocity) to achieve full cooperation^1–3^; and recent studies offer various approaches to estimate strategy distributions based on experimental data^4–7^. Indirect reciprocity may be of positive impact, both in theory and experiments^8–14^. Options of punishment^15–20^ and reward^21–23^ may also greatly improve cooperation in experimental and theoretical studies, amidst the strong reciprocity debate^24,25^. We study the issue of cooperation using prisoner’s dilemma (PD) as the basic game within the general assortative matching (AM) framework, as e.g. studied in^26–29.^ Note that^30^ allows for type recognition to generate AM effect, and public goods games with endogenous group formation may induce assortative outcomes in favor of cooperation^31,32^.

Assortative matching has been identified as a universal principle in human and non-human ecologies as a mechanism for promotion of pro-social behavior. Ref.^28^ illustrates stylized facts and anecdotal stories in human societies and points out characteristic behavioral features underneath a functioning condition of assortative matching. In general, honest and trustworthy persons often display non-imitable behavioral and biological traits or labels, which enable people to be selective in partner choice for joint endeavor such as the PD game, resulting in like-minded people to be more likely matched that it would be random that reflects the saying “birds of a feather flock together”. In this environment, cooperation has a chance to proliferate for the betterment of society.

References^33,34^ provide a thorough discussion of the common evolutionary models of assortative matching. Consider the generic PD game with T > R > P > S, where one player’s payoff is R, P, T, or S, if both cooperate (C), both defect (D), the player is the exploiter, or he the sucker, respectively. Consider a population of *x *∈ [0, 1] cooperators and 1 − *x* defectors. Define $a(x)=P(C|C)-P(C|D)$ as the *index of assortativity*, where *P*(*X*|*Y*) denotes the conditional probability that a type-*Y* player expects to be matched with a type-*X* player. Assume replicator dynamics for population change $\dot{x}$, it is perfectly aligned with the sign of fitness difference between C and D types, $\delta (x)={\bar{\pi }}_{C}(x)-{\bar{\pi }}_{D}(x)$, given the population state *x* and the index $a(\,\cdot \,)$.

Depending on application environments, various forms of index of assortativity are conceivable as detailed in^34^. For the most simple case, it can be constant with *a*(*x*) = *a*. The equilibrium analysis is straightforward in this case. With *a* sufficiently high, cooperation may survive in the long run, either in form of a stable mixed population equilibrium or as one of the stable pure population equilibria. However, envision the physical realization of a real-world sorting mechanism, it is reasonable to assume that individuals need to spend some small but positive search cost in pursuit of a proper match. In fact, the existence of such cost is exactly the very reason for the reasonable assumption of imperfect assortativity *a*(*x*) < 1. This implies that in the pure population states, the matching result must be equivalent to random matching due to zero likelihood to meet the lacking type, i.e. *a*(0) = *a*(1) = 0, where only defection prevails in the long run.

Fortunately,^34^ demonstrates that non-constant AM that satisfies *a*(0) = *a*(1) = 0 exists, such as in the so-called *stranger-in-the-night* model, where cooperation survives in form of a locally stable mixed population equilibrium.

Stranger-In-The-Night Model: Imagine each of two different types has distinctive appearance characteristics that are not perfectly recognizable in the night when strangers are supposed meet and decide whether go home with each other. Let *s* and *m* with $0 < m < s\le 1$ denote the match success rates if the randomly encountered counterpart is of the same and different type, respectively, then the resulting index of assortativity is

$$a(x):=\frac{xs}{xs\,+(1-x)m}-\frac{xm}{(1-x)s\,+xm}=\frac{x(1-x)({s}^{2}-{m}^{2})}{x(1-x){(s-m)}^{2}+sm}.$$

Straightforward calculation yields *a*(0) = *a*(1) = 0. Note that ${\max }_{x}a(x)=a(1/2)=$ $(s-m)/(s+m)$, i.e. the assortativity effect is strongest when C and D have equal shares in the population. With *δ*(*x*) properly spelled out, it is obvious that *δ*(1/2) > 0 with *m* sufficiently small.

In this specific model, *δ*(*x*) = 0 has three solutions $\{0,{x}_{{\rm{\min }}},{x}_{{\rm{\max }}}\}$, $0 < {x}_{{\rm{\min }}} < {x}_{{\rm{\max }}} < 1$, where 0 and *x*_max_ are locally stable equilibria with respective basins of attraction (0, *x*_min_) and (*x*_min_, 1]. The general insight from this theoretical discussion is as follows. While pure defection is the only stable population equilibrium in RM, AM potentially may admit additional stable equilibria with a higher share of cooperators, depending on the specifics of the mechanism. Moreover, in the latter case, the initial state of population is crucial at determining whether the dynamics converges to the bad pure defectors equilibrium of x^*^ = 0 or some better ones such as x^*^ = *x*_max_. This, henceforth, opens up the gate for culture, social conventions or state actions to be shaped and constructed in a way so as to positively affect either the effectiveness of AM mechanism, for example by increasing *s* or decreasing *m* in the strangers-in-the-night model above; or the initial state of aggregate pro-social propensity, for the long-term proliferation of cooperation.

The main objective of the study is to design an experiment with an AM setup to investigate the effect of exogenous shocks to the initial population state of cooperation in PD.

## Methods

### Experimental design

References^35,36^ and most intriguingly^37,38^ show experimental evidence that human subjects recognize others’ behavior types with substantial accuracy. When playing the PD game, the aggregate effect of such recognition on the outcome is significantly positive and displays the AM property. We believe one way to describe the situation is the saying, “you are what you do”. As argued by^28^, physiological cues or reaction-circuits get hard-wired for repeatedly practicing certain behavior, so that imitation is hard to do. In words by Confucius, pretenders always get exposed, and thus it is a better strategy to be consistently pro-social even when nobody is watching, as in the following quote.

“There is no evil to which the mean man, dwelling retired, will not proceed, but when he sees a superior man, he instantly tries to disguise himself, concealing his evil, and displaying what is good. Yet, the other beholds him, as if he saw his heart and reins;–of what use is his disguise! This is an instance of the saying–‘What truly is within will be manifested without.’ Therefore, the superior man must be watchful over himself when he is alone.”–*Great Learning* 6:2^39^.

Based on this, we ask the question that, suppose the type recognition part for assortative matching is solved exogenously, would human subjects indeed make use of it so as to improve the general level of cooperation in PD?

We have three treatments. Every treatment has 5 groups of 14 real subjects. A typical session consists of 2 or 3 separate games. The treatments differ in Game 2 where subjects play 25 rounds of a given PD game. As a control, we did a random-matching treatment (RM) with 25 rounds and information about subjects’ personal experience. With (T, R, P, S) = (12, 8, 3, 1), we use a PD game that makes defection very attractive, i.e. it is submodular^40^.

In the treatment called weighted-history (WH), subjects know they will be assigned a T-score according to their behavior in the past 5 rounds, which is calculated by first identifying decisions {C, D} as {1, 0} respectively and weighting the history with the Fibonacci numbers (5, 3, 2, 1, 1) that favors the nearer past. This makes T-score a number between 0 and 12 for our purpose. Ties are randomly broken. The partner’s T-score is not known, but the matching principle is where all people in the group are first sorted by their current T-score, with ties broken randomly, and players are matched pair-wise according to their ranks from low to high. So, they know that their new partner is a neighbor with regard to this ranking, potentially with similar behavior in the past 5 rounds. As to available information, subjects have only on record their own personal experience, from which the T-score is calculated. This basic treatment is called WH. (More discussion of T-score and WH treatment can be found in^41^) Note that our design meets the sensible condition *a*(0) = *a*(1) = 0 for AM. With WH as surrogate for AM now, our motivating questions can be summarized in the following hypothesis.

### Hypothesis

First, the WH setup is strong enough to generate significantly higher level of cooperation than RM does. Second, by adding cooperative dummies, cooperation may prevail in more groups and at an even higher level.

References^41,42^ offered an initial answer to the first question. WH had certain level of success in some societies compared to RM. But in some others, the group dynamic led to almost all subjects into pure defectors, with even higher speed. How to get people out of the dreaded absorbing state of pure defection? For this purpose, we modify the initial WH treatment with the following feature: Subjects are aware that there are 14 real players and 2 computer dummies in the society, with exactly the same rules as under WH applied now to a 16-people society; subjects are informed that the dummies are programmed to play one action persistently through all 25 rounds, either C or D, without being aware that only C-dummies are employed. (We design this uncertainty to avoid the objection of too strong experimenter-suggestive framing towards cooperation. The possibility of “bad” dummies may induce some repelling effect to keep people away from always-defection, in WH. But our dummies are always-C in implementation. So, in some way, this design induces both *sticks* and *carrots* to attract people away from defection and towards cooperation.) We label this treatment WHc. For real world motivation, the presence of pro-social dummies can be motivated by any political and social system that promotes virtue such as via religion or state propaganda, as long as we expect them to be effective at raising the initial stock of pro-social inclination in society.

In Game 1 of each session, they first play the same PD game 5 rounds with random matching, but *without* feedbacks. Here, subjects are virtually asked to reveal their (mixed strategy) inclination to cooperation in a one-shot PD game, which can be later linked to their subsequent treatment behavior. Besides, it serves as a sampling-bias test, to ensure comparability of data across treatments. Subjects did not get any information about the next game that came after the end of the current one.

One justified concern against the design is whether subjects’ behavior is already stable in merely 25 rounds of play. Though, for reasons of subjects’ fatigues and boredom, the length of experiment as well as the number of rounds should be kept limited, we have repeated WHc with a clean slate, called WHc3 henceforth as Game 3 in the WHc sessions, to test behavior stability. In particular, it might offer insight as to the value of applying more complicated learning models to the available data sets.

Details of the instructions can be found in the appendix. We did many quiz to make sure that subjects understand the rather complex matching scheme. For all three treatments RM, WH and WHc, we recruited a total of 210 students of various majors at National Chengchi University in Taiwan to collect data.

## Results

### Data analysis

Let us start the analysis by stating that across the treatments there is no sampling bias as measured in cooperation rate, P(C), in Game 1. Treatment averages range from 0.36 to 0.41, without significant differences (Kruskal-Wallis test, p = 0.7712; more details in Table A1). Note that for a quasi one-shot PD game, these numbers are similar to those in the literature^43,44^.

**Figure 1:**
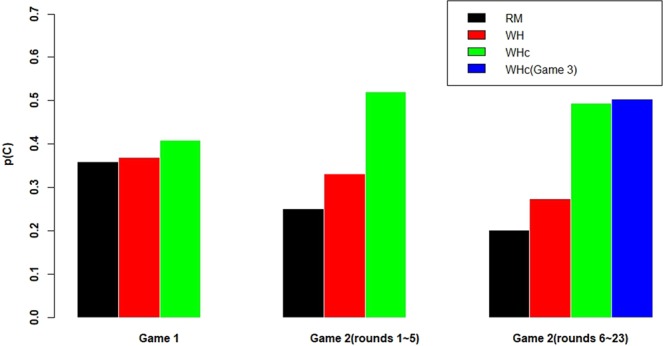
Treatment–level cooperation rate in different phases. WHc has significantly higher C rate than WH and RM. WH bifurcates around the more homogenous group performances in RM.

Figure 1 summarizes the average rate of cooperation for the first 5 rounds and the rounds 6–23 of Game 2 in each treatment separately, as well as Game 3 in WHc treatment (WHc3), besides that for Game 1. The last two periods in Game 2 and 3 are dropped, due to end-game behavior (as discernible in Fig. A1). The first 5 rounds of Game 2 are treated separately, because we suspect some learning effect before the matching score T can reach the maximum 12 for anybody.

Although player behavior in Game 1 does not differ across treatments, their behavior is quite different in Game 2. For both rounds 1–5 and 6–23, the cooperation rate is the smallest for RM with slight increase to WH and a big jump to WHc (and WHc3). First of all, Wilcoxon test shows that RM and WH are not significantly different in median (p = 0.1425 and p = 0.1425), for both rounds 1–5 and rounds 6–23 respectively. However, Ansari-Bradley test shows that RM and WH are different regarding group variations with p = 0.011 for t = 6–23. In fact, the ranked session average C rates are (0.139, 0.179, 0.214, 0.218, 0.258), (0.083, 0.135, 0.333, 0.405, 0.409), (0.377, 0.421, 0.488, 0.571, 0.615), for RM, WH, WHc respectively. Note, Ansari-Bradley-test for t = 1–5 yields p = 0.3902. Put together, this indicates that the bifurcation effect in WH solely stems from later dynamics in t = 6–23This outcome bifurcation result is consistent with (the first part of) our Hypothesis that is based on a regular AM model.

Second, WHc displays higher cooperation level than both RM (p = 0.0079 and p = 0.0079) and WH (p = 0.0119 and p = 0.0318). Thus, the insertion of dummies is highly effective, confirming (the second part of) our Hypothesis. In Appendix, Table A2 shows more group-level numbers in Game 2 (t = 6–23), while Fig. A1 shows the time trend of p(C) for another visual display of treatment difference.

**Figure 2:**
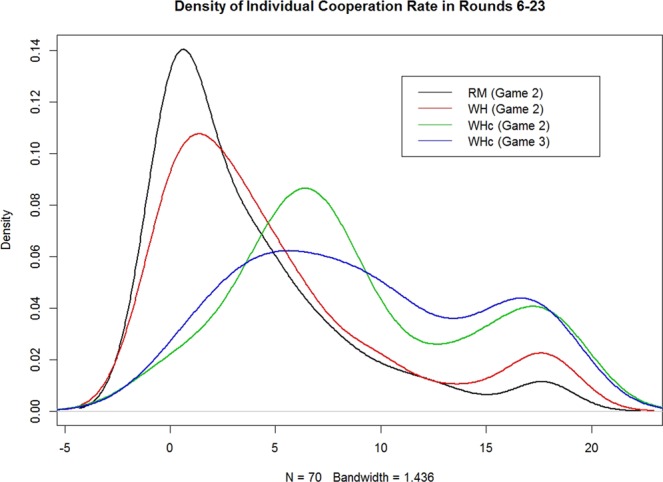
Smoothing Distribution of Individual Cooperation Rate p(C) for Game 2, t = 6–23. WHc > WH > RM on population share around the high peak. In addition, WHc repositions the lower peak to the right, which reflects the repelling effect expected from the design. (Created using the freeware “R”, with the command “kernel” at the default setting).

So far, the analysis is on group level. What kind of changes do treatment variations bring to the individual level of behavior? Looking at the cumulative distribution functions (cdf) of individuals’ cooperation rate over rounds 6–23 in Game 2, as illustrated in Fig. A2 based on Table A3 with 70 samples for each treatment, it is visible that the cdf curve of RM is always on the top of, i.e. stochastically dominates, WH, and that of WH is on the top of WHc/WHc3. Figure 2 shows the corresponding density function (pdf), based on kernel smoothing^45^. RM and WH both have two peaks, for strong inclination of playing either cooperation or defection. The pdf curves of WHc and WHc3 apparently shift to the right, with much reduced probability in choosing all defection and much higher probability in choosing all cooperation. This can be also seen in Table A4.1, where the number of players choosing C 12–15 and 16–18 times is obviously higher for WHc and WHc3. Note also that WHc3 seems to move the peaks in WHc further apart, which indicates minor learning effect towards more behavior bifurcation. Keep in mind that individual level behavior in Game 1 is not significantly different across treatment (χ^2^-test, p = 0.2704; see Table A6.2), which further confirms the fact of no sampling bias in our study.

**Figure 3:**
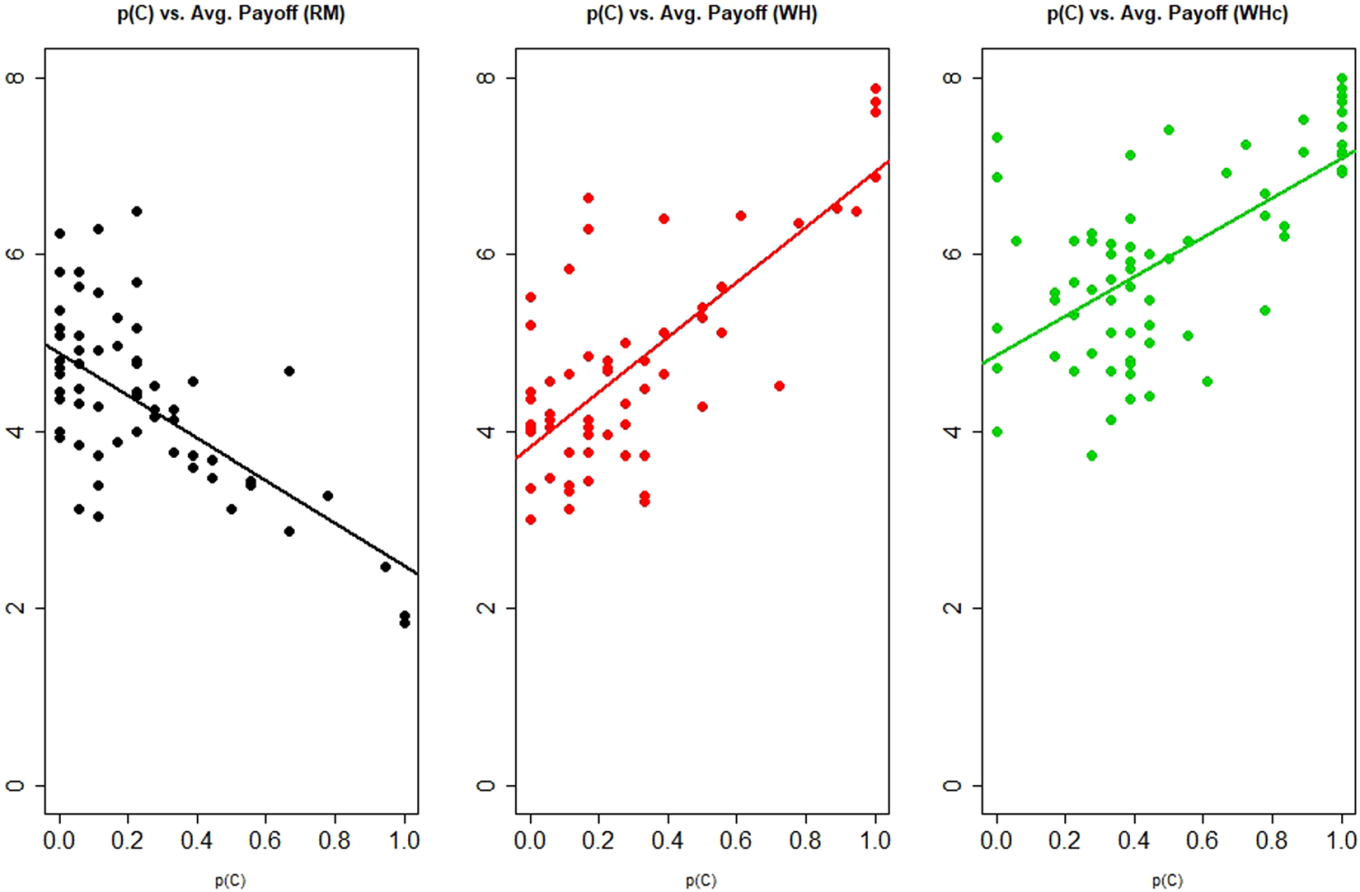
Scatter plots: Payoff vs. cooperation rate. AM induces positive correlation between cooperation and payoff.

Figure 3 is a scatter plot that illustrates how individual C-rate is related to one’s avg. payoff, t = 6–23. We see that it is clearly negatively correlated in RM, as expected, but positively correlated in WH and WHc, with correlation coefficient being −0.5834, 0.7752 and 0.6582 respectively.

All in all, we conclude that WHc moves all people in society to be more cooperative! Given the dummies, the always-D state appears more repelling while the always-C state more attracting, as predicted in the design discussion. WH, in comparison, seems less effective at rattling people from lethargy. At times it generates faster decline towards the all-D state than even in RM, among a sub population. In other words, evolution of cooperation a la cultural evolution mechanisms in WH as argued in^41^ might take extremely long to reach a satisfactory level of cooperation, in comparison to WHc.

### Assortative matching effect

Real subjects often change behavior during the course of experiment, so that the binary type assumption in the theory model is too coarse. An approximate measure for assortativity can be obtained by looking at the frequency of realized outcomes, CC CD (or DC) and DD within the matched pairs, in comparison with the hypothetical random-matching distribution that can be straightforwardly calculated using the observed cooperation rate P(C). In RM, they are identical, meaning there is no assortativity effect as predicted. In WH and WHc, on the other hand, the observed CC and DD frequencies are much larger, in other words CD much smaller, than the hypothetical random matching values. We separate the match outcomes into CD vs. non-CD (i.e., CC & DD) and use χ^2^ test to check if they fit random matching. The p-values for RM, WH, and WHc are 0.5636, 0.0000, and 0.0000, where p-value < 0.05 indicates non-random matching (also see Table A5 and Fig. A3).

### Micro-level behavior determinants

Without information feedback, Game-1 behavior indicates one’s initial propensity to cooperation in a one-shot PG game. How would mature individual treatment responses (rounds 6–23 behavior) depend on their Game-1 behavior? Also, though maybe still in a phase of testing the field, subjects may reveal their understanding of the game rule in their initial actions in the first 5 rounds in Game 2. Table A6.1 summarizes all relevant correlation coefficients for this consideration, which are significant and positive in all treatments, indicating general behavior inertia.

**Figure 4:**
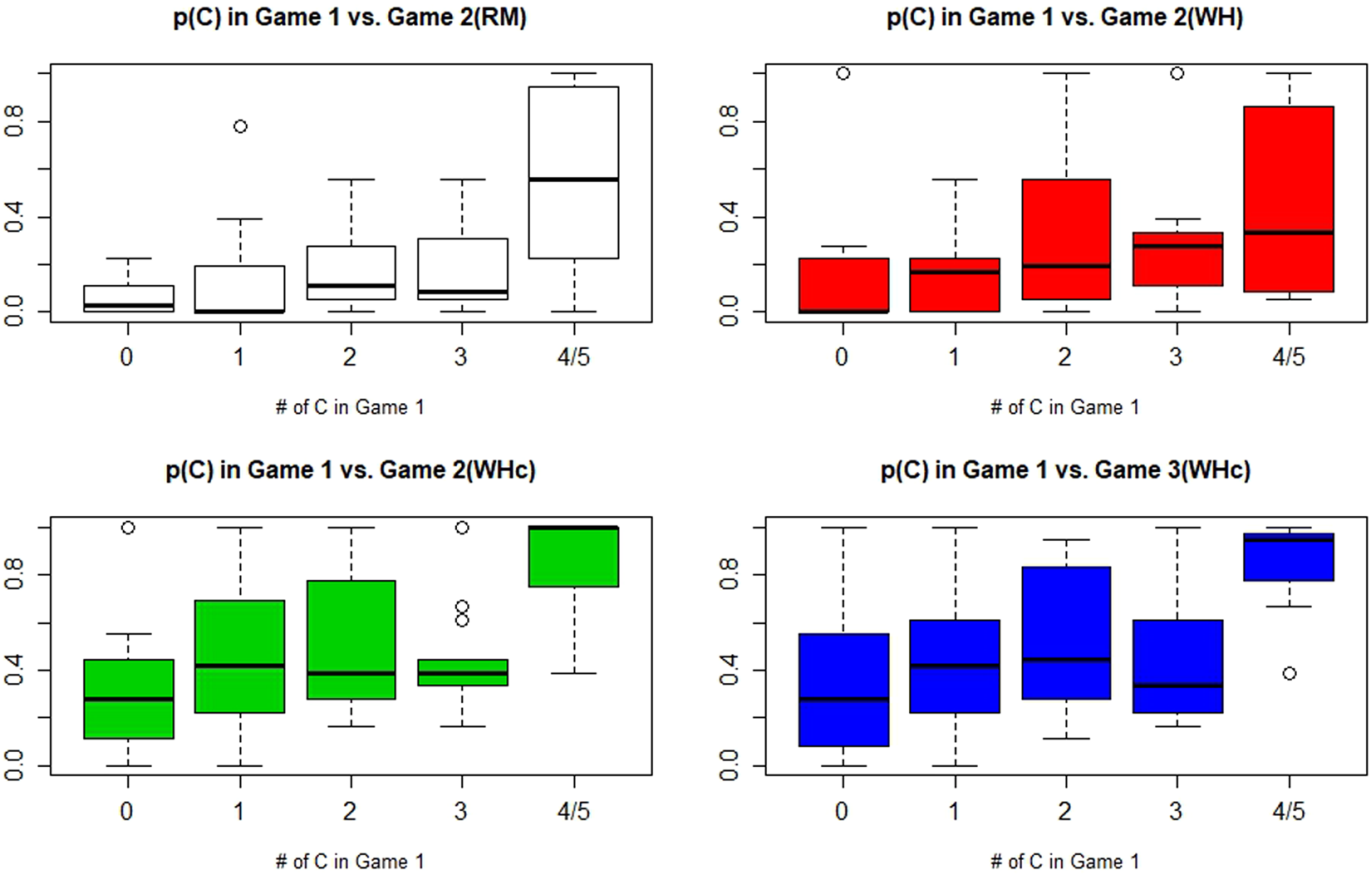
Cooperation rate, Game 1 vs. Game 2 (t = 6–23). WHc > WH/RM for all initial individual propensity to cooperation, i.e., the attracting effects towards more cooperation uniformly affect all initial types of one-shot PD.

Figure 4 shows the boxplots of P(C) in Game 2, given the choices of Game 1. Apparently, more cooperative Game-1 players are also more cooperative in Game 2. Most noteworthy is however that for each level of Game-1 cooperation, the associated Game-2 level of cooperation is much higher in WHc than in both RM and WH, which is significantly so for players with #C < 4 in Game 1 (p = 0.0086, 0.0101, 0.0019, and 0.0071 for the groups of #C = 0, 1, 2, and 3 players; Kruskal-Wallis test). Note that, in RM, individuals with #C ≥ 4 in Game 1 still played very cooperatively in Game 2, which simply reflects their initial inclination to cooperation. (The Wilcoxon-test yields p = 0.0040 and p = 0.4821 comparing WHC with RM and WH, respectively.) For these initially strong cooperators, the bifurcating dynamics in WH in fact made some of them more opportunistic, while in WHc they remained the same good people throughout.

Thus, we conclude that the total positive effect for WHc can be found in the push-and-pull mechanics: repelled by the sticks of being stuck with bad dummies and attracted by the prospect of more likely matched with the good dummies as well as other real subjects that strive for the same.

Figure A4 in Appendix reveals how the initial treatment responses in t = 1–5 affect the mature responses in t = 6–23 within Game 2. For all treatments, the correlation coefficients between t = 1–5 and t = 6–23 in Game 2 are all larger than those between Game 1 and t = 6–23 in Game 2 (Table A6.1). This suggests that many players’ behavior likely became stable already at the very beginning of Game 2.

Conspicuously, WHc exerted a strong effect on the initially pure defectors (#C = 0, t = 1–5), and made them cooperate at the 40% rate later in t = 6–23. This seems again to be the speculated “repelling” effect due to uncertainty about dummy types. Subjects conceivably may falsely attribute their frequent encounters with defection as being constantly matched with the bad dummies, and grudgingly yield to the reality of no other choice but joining the more cooperative fellows in the society.

### Regression analysis

As Fig. A5 illustrates, past aggregate actions are highly correlated with cooperation rate for all treatments in this study. Following^41^ where the best fitting regression for P(C) in Game 2 (t = 6–23) for RM and WH is a 3^rd^-order polynomial in the matching score T, the basic regression equation we employ is as follows, where “other terms” vary in pursuit of best fitting.

$${\rm{logit}}(p(C))={\beta }_{0}+{\beta }_{1}T+{\beta }_{2}{T}^{2}+{\beta }_{3}{T}^{3}+{\rm{other}}\,{\rm{terms}}$$

Among other terms, it seems that using Game-1 C-rate as the additional determinant yield robust results for all treatments, where *β*_0_ is clearly higher in WHC than for RM and WH. In addition, replacing Game-1 C-rate with #C = 1,4,5 in Game 2 (t = 1–5) yields slightly better result for WHc. That these subjects later played C to relatively higher rates reflects the *repelling* and *attracting* effects of the pure-action types generated by our dummy design. Since the regression results generally confirm the previous observations without much crucial new insight, we refer interested readers to the appendix for more detailed discussions. For WH and RM, we also did some preliminary learning model analysis. In general, aside from RM and some convergent groups in WH, the standard reinforcement and EWA models are rather bad, mostly outperformed by static fitting of C-rate or a more sophisticated variation of two-phase best fitting. And comparison between WHc and WHc3 suggests that the patterns of behavior are rather stable after 25 rounds. Note, we also made subjects play another game for 25 rounds in all the treatments, but they were designed more like pilots for later treatments rather than specific robustness tests as in WHc sessions, and was thus omitted here.

## Discussions

In society where AM is featured as a guiding behavior doctrine, state promotion of virtue role models can be effective at fostering cooperation. As documented by the Confucian classics^39,46^, the Confucian culture explicitly features AM. In fact, for the past 3000 years Chinese rulers of all dynasties continually implemented meticulous systems of honor conferring to promote virtue role models, whose selection used to be a major administrative task for local magistrates^47^. Thus, Weber’s^48,49^ contention that the lack of transcendence in Confucianism be prohibitive to China’s modern economic prospect may have missed a salient factor that is AM, which may constitute an effective system alternative at promoting trust in society. The so-called Asian, and Chinese, growth miracle may indeed be deep rooted in the Confucian cultural heritage^50,51^.

Given such cultural and historical evidence for assortativity as facilitator for cooperation, the common two-type evolutionary model is indeed still primitive. Before more advanced models can be properly developed, our WH design to experimentally investigate the AM effect serves as an early attempt to find empirical evidence in support of the theoretical cooperation-promoting predictions, encouraged by early findings on type recognition^35–38^.

If via state propaganda and incentivized promotion the ‘economic men’ or opportunist cooperators are made aware of the existence of a small number of virtue men, i.e., unconditional cooperators, we conjecture that they would be better motivated to be more cooperative in order to have higher chance to partner up with the virtue men as a result of the underlying AM mechanism. In fact, if this motivation is effective, there may be enough induced high-cooperating opportunists who serve as sufficiently good surrogates for the virtue men, for the purpose of meeting a person with high cooperation inclination. Note, however, it is theoretically also possible that the injection of exogenous virtue men like those resulting from rewarded state promotion may ex ante not have any effect within the remaining population. E.g., in groups in the WH treatment that exhibit stable bifurcation into all-C and all-D players, injecting additional all-C players will not change anything among the existing population. But there is hope that during the transition of learning how to play the field, the existence of exogenous virtue men may exert enough dynamic pulling effect toward more cooperation from the opportunists. In the end, our WHc with dummies is successful!

In this paper, we showed experimentally that the designed AM setup combined with exogenous cooperative dummies that may be interpreted as capturing the effect of institutional promotions of virtue role models can significantly increase the success of cooperation overall, in contrast to^41^ where without such dummies success of AM occurs only in a subpart of groups. As predicted by the model, we observe in WHc a fundamental shift towards a population with more cooperators and fewer defectors compared to both WH and RM (Fig. 2). As indicated by Fig. 4, while WH’s relative success over RM may be tracked back to better motivating subjects with mid-level initial cooperativeness to not drift down to pure defectors, WHc is able to mobilize even the initially most defective and cooperative subjects. Data seem to suggest that under WHc pure defection and cooperation exert a repelling and a pulling force, respectively. Note, as implicitly in our stated empirical hypothesis, the effects of both WH and WHc are consistent with a theoretical model such as stranger-in-the-night where the initial population state is crucial.

Future research may benefit from development of more sophisticated search and matching models with assortative features, such as real-world network formation. As a historical stylized fact, for example, the same-year entrants who passed the Chinese imperial national exam would traditionally keep a close bond the rest of their career, likely for both individual benefits and as means to encourage their pro-social behavior in fulfilling the magistrate duties. In an experiment with voluntary network choices^52^ observes AM property in the data. However, the success of cooperation there may be due to the strong scale effect via adding more connections. Note^53^, also confirm the idea of^54^ in a rigorous model of indirect evolution of preference and show that the index of assortativity determines which Kantian type survives in the stable single-type population equilibrium. Finally, besides assortativity and the settings mentioned in Introduction, various settings have also been discussed for promotion of cooperation such as in^55–58^, among others.

### Ethics

All methods were carried out in accordance with relevant guidelines and regulations. All experimental protocols were approved by the ethics review board of RCHSS, Academia Sinica. Informed consent was obtained from all subjects upon presentations of the experiment instructions.

## Supplementary information

Cooperation in an Assortative Matching Prisoners Dilemma Experiment with Pro-Social Dummies Supplementary Material
