# Supplementary material for: Cooperation in an Assortative Matching Prisoners Dilemma Experiment with Pro-Social Dummies
Source: Sci Rep. 2019 Sep 20;9:13609. doi: 10.1038/s41598-019-50083-6 (PMC6754460; doi:10.1038/s41598-019-50083-6)
Supplement: Supplementary file 1 — Cooperation in an Assortative Matching Prisoners Dilemma Experiment with Pro-Social Dummies Supplementary Material [file 41598_2019_50083_MOESM1_ESM.pdf]

Supplementary Material

Cooperation in an Assortative Matching Prisoners Dilemma Experiment with Pro-Social Dummies

Chun-Lei Yang[1] and Ching-Syang Jack Yue[2]

[1] Economics Experimental Lab, Nanjing Audit University, 86 Yushanxi Road, Nanjing 211815, China; ycl@nau.edu.cn

[2]Department of Statistics, National Chengchi University, Taipei, Taiwan, ROC; csyue@nccu.edu.tw

Appendix 1: Experiment Instructions

General Instruction

You are about to participate in an economic experiment on multi-person interactive decisions. The study is funded by the NSF and Academia Sinica.

Basic experimental rules

1. Please read these instructions completely and carefully. Keep quiet during the experiment and do not contact fellow subjects under any circumstances. Any question shall be raised to the experimenters individually.
2. The experiment is anonymous and you will get an ID card before the start.
3. Please do not do anything on the PC that is irrelevant to the experiment.
4. You will be paid off anonymously after the experiment ends, in exchange for the ID card you hold.
5. Please return all instructions you have received at the end of the experiment.
6. To help us collect reliable data, please do not talk about this experiment in the next two weeks with those who have not participated. Thank you for your cooperation.

Generic decision rules

There are 14 subjects who participate in this session. Each session contains several separate 2-person games of length from 5 to 25 rounds. Below is a demo window for what you will see on the PC in the experiment. (Correct payoffs numbers will be

given during the experiment.)

In each round, when the decision bars become highlighted, you can start to make a decision between A and B. An example is shown in these printed instructions to tell you how to find out what you and your counterpart will receive as a consequence of your decisions.

At the end, you will be paid off the sum of the payoffs you get in each round, plus a 50NT show-up fee. For experimental purposes, in some of the games you will not be informed immediately of the action of the other person and thus of your own payoff there. On the upper right of the window, you will see your account balance for all the other games/rounds played, including the 50 NT show-up fee. At the end, your final balance will be updated, with all the payoff and action information previously withheld.

Note that the computer rematches the participants into pairs after each round. The matching procedure changes during the session. You will be informed about the details in special written instructions accordingly. Wait for the experimenter's further instruction.

During the session, you will find the following information on the right of the window: (1) Your own choices so far; (2) Your counterpart's choices; (3) Your own payoffs in the previous rounds; (4) Other information and instructions. For part of the experiment, you will not get information about (2) and (3) right away, but you will find "?" instead. This information will be given at the end of the session.

Special Instructions

Random Matching: 5 rounds no feedback (Games 1 and 3)

In this part, there will be five rounds. At the beginning of each round, subjects will be randomly paired to play the game you will find on the window. This means that you have the same chance to meet any of the other 13 subjects in each round, independent of what has happened so far. For experimental purposes, you will be informed neither of your counterpart's action nor of your own payoff, at this time. You will receive the relevant information at the end of the session and your total final payoff will be updated accordingly.

Random Matching: 25 rounds (Game 2)

In this part, there will be 25 rounds. At the beginning of each round, subjects will be randomly paired to play the game you will find on the window. This means that you have the same chance to meet any of the other 13 subjects in each round, independent of what has happened so far.

Weighted-history Correlated Matching [*with Dummies*] (Game 2)

(Note, in what follows parts in […] and italics are additional explanations for WHc compared to the Instructions for WH.)

In this part, there will be 25 rounds. [*Two computer dummies join the session. They are programmed to take one fixed action, either A or B, throughout whole 25 rounds*.] At the beginning of the first round, all [*16*] subjects will be randomly paired to play the game you will find on the window. For the other rounds, the matching procedure takes into account what the subjects have done in the previous five rounds in the following form.

**Rd**	**Last 5 decisions**	**T= T_1 + T_2 + T_3 + T_4+ T_5**
1	none	0
2	A	0 = 0
3	BA	5 + 0 = 0
4	BBA	5 + 3 +0 = 8
5	ABBA	0 + 3 + 2 +0 = 5
6	BABBA	5 + 0 + 2 + 1 + 0 = 8
7	ABABB	0 + 3 + 0 + 1 + 1 =5

<u>Step 1: Calculation of the sorting score (T)</u>

At each round, if your choice in the previous round is B you will get a score T1=5. If B is your choice in the 2nd previous round, you get a score T2=3. If B is your choice in the 3rd previous round, you get a score T3=2. If B is your choice in the 4th previous round, you get a score T4=1. If B is your choice in the 5th previous round, you get a score T5=1. Otherwise, i.e. if your choice is A in the n-th previous round, your score is Tn=0. The total sorting score T = T1+T2+T3+T4+T5. For the very first 5 rounds of this part, T is calculated with the previous rounds' actions only. By definition, then, all

subjects start this part with the same T=0.

For illustration, assume that somebody's choices in round 1-6 are ABBAABA.

For example, in round 7, T=5 is calculated in the following way. Since A is the choice in the previous round (round 6), T1=0. B in the 2nd previous one (round 5), T2=3. Etc.

<u>Step 2: Matching according to the sorting scores</u>

At the beginning of each round, all subjects will be ranked according to their ranking scores T and be paired with their neighbors, subsequently from top to bottom.

Subjects with the same ranking score T will be ranked randomly among themselves.

For example, if there were four subjects a, b, c and d in the experiment (in reality 14) with the ranking scores 5, 5, 0 and 8 accordingly, then they would be ranked either as {d,a,b,c} or {d,b,a,c} with equal chance. Then, we would end up with the matching result of either {(d plays with a), (b with c)} or {(d with b), (a with c)} with equal chance accordingly.

Note that this matching procedure ignores the actions earlier than five rounds ago.

Also you can at any time find out in the window on the right side of your monitor about your own previous choices, your previous counterpart's choices, your payoffs, your account balance, and your current ranking score T.

<u>Test</u>

1. If subject a's choices in the first 4 rounds are ABBA, then a's ranking score in the 5th round is: (1) 0, (2) 1, (3) 3, (4) 5.
2. If a's choices from round 3 to round 7 are ABABB, then his ranking score in the 8th round is: (1) 0, (2) 1, (3) 5, (4) 9.
3. If six subjects a, b, c, d, e, f participate in the experiment and, at some round, have the ranking scores of 8, 12, 10, 12, 5, 0, then a can be potentially matched with: (1) only b, (2) only c, (3) b or c, (4) b or d.
4. If six subjects a, b, c, d, e, f participate in the experiment and, at some round, have the ranking scores of 8, 12, 10, 10, 5, 0, then a can be potentially matched with: (1) only b, (2) only c, (3) c or d, (4) b or d.

Answers: 1. (4); 2. (4); 3. (2); 4. (3).

Weighted-history Correlated Matching with Dummies (Game 3)

The same as for Game 2!

Appendix 2: More Supplementary Data

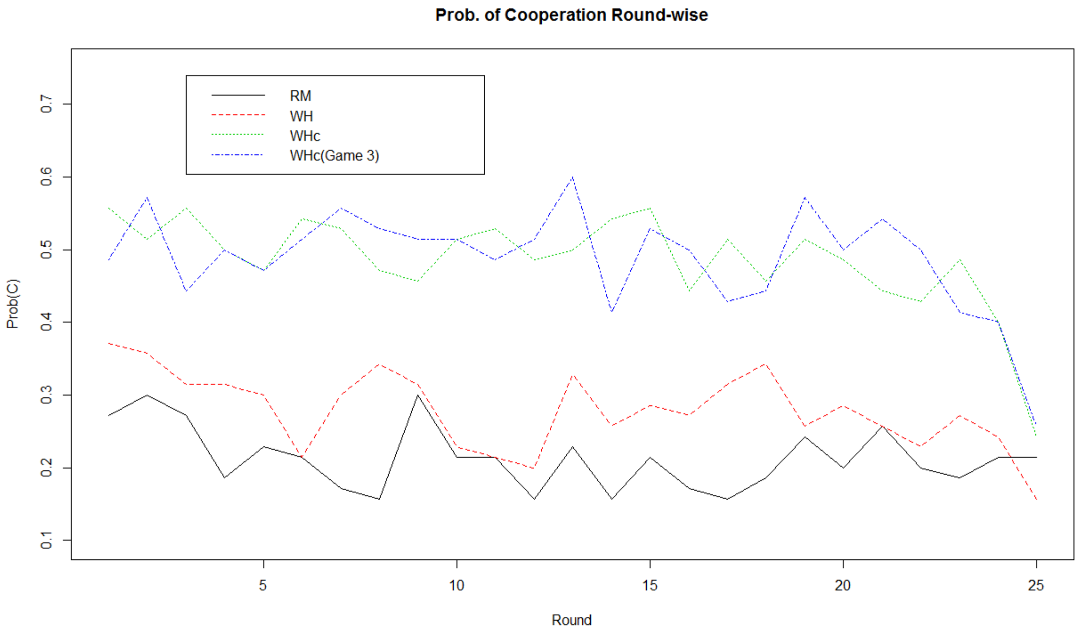

Figure A1. Time-Trend of Cooperation rate in Game 2/3

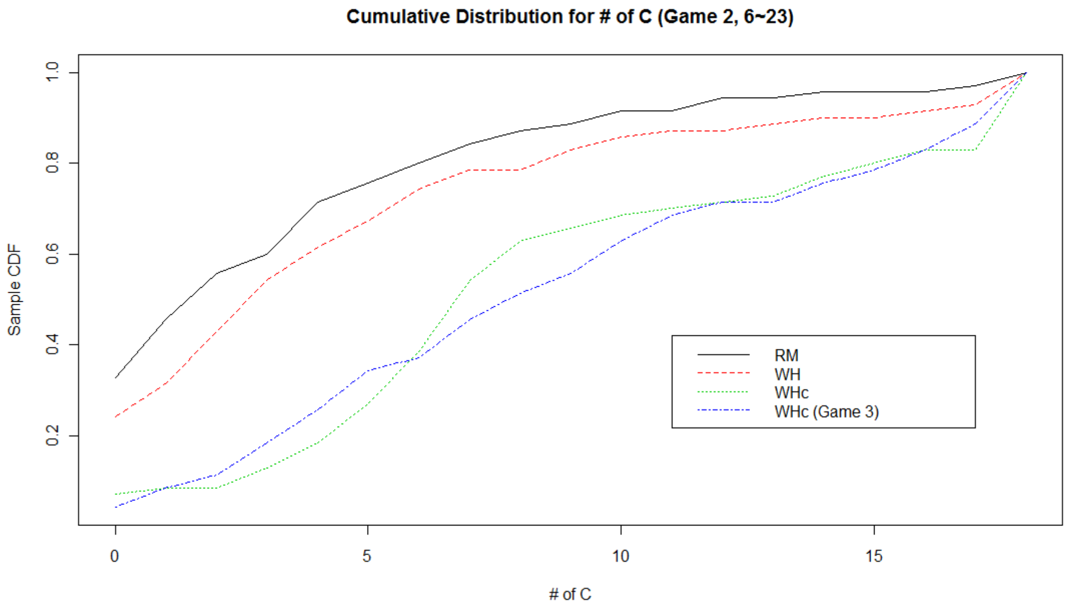

Figure A2. Cumulative Distribution of C-rate of single Players (Game 2/3, 6-23)

Table A1. Group avg. rate of Cooperation in Game 1

	RM	WH	WHc
1	0.286	0.371	0.543
2	0.443	0.257	0.243
3	0.343	0.314	0.357
4	0.371	0.471	0.529
5	0.357	0.429	0.371
Avg.	0.360	0.369	0.409
Std.	0.057	0.086	0.126

Table A2. Group level summary over rounds 6-23 (Game 2 or 3)

Treatment	Group	p(c)	p(sw)	Av-r	r-ratio
	**1**	0.214	0.174	4.468	0.288
	**2**	0.258	0.254	4.599	0.779
**RM**	**3**	0.139	0.159	3.925	0.523
	**4**	0.179	0.131	4.171	0.565
	**5**	0.218	0.226	4.433	0.509
	**avg**	**0.202**	**0.189**	**4.319**	**0.533**
	**std**	**0.045**	**0.050**	**0.270**	**0.175**
	**6**	0.409	0.099	5.194	1.617
	**7**	0.083	0.143	3.567	0.446
**WH**	**8**	0.333	0.262	5.016	0.809
	**9**	0.405	0.349	5.357	0.927
	**10**	0.135	0.194	3.897	0.538
	**avg**	**0.273**	**0.210**	**4.606**	**0.867**
	**std**	**0.154**	**0.099**	**0.815**	**0.463**
	**16**	0.615	0.298	6.409	1.042
	**17**	0.488	0.269	5.746	1.115
**WHc**	**18**	0.377	0.391	5.635	0.608
	**19**	0.571	0.244	6.365	0.804
	**20**	0.421	0.420	5.675	0.674
	**avg**	**0.494**	**0.324**	**5.967**	**0.849**
	**std**	**0.100**	**0.077**	**0.387**	**0.223**
	**16**	0.571	0.393	6.343	0.824
	**17**	0.550	0.346	6.196	0.860
**WHc**	**18**	0.461	0.386	5.986	0.626
**(Game 3)**	**19**	0.496	0.189	5.779	1.324
	**20**	0.432	0.310	5.614	0.851
	**avg**	**0.502**	**0.325**	**5.984**	**0.897**
	**std**	**0.059**	**0.083**	**0.297**	**0.257**
**p-value of Wilcoxon test**	WH vs. WHc	.0283	.0758	.0090	.4647

Note: The numbers with light background indicate significance at 0.10 and numbers with darker background are significant at 0.05. p(c) and p(sw) denote cooperation rate and switching rate in Game 2 or 3. Av-r = average payoff/reward achieved; r-ratio =

[avg. payoff playing C]/[avg. payoff playing D]

Table A3. Individual C frequencies (Rounds 6~23)

Treatment	Group	# of C's													
RM	1	0	0	0	0	0	0	1	2	3	5	6	7	12	18
	2	1	1	1	2	2	2	3	4	4	4	5	7	12	17
	3	0	0	0	0	0	0	1	2	2	3	4	6	8	9
	4	0	0	0	0	0	0	0	0	1	2	6	8	10	18
	5	0	0	0	1	1	1	4	4	4	4	5	7	10	14
WH	6	0	0	0	0	1	2	3	3	10	14	16	18	18	18
	7	0	0	0	0	0	0	0	1	2	2	3	3	4	6
	8	0	0	2	2	3	4	5	5	6	6	6	9	18	18
	9	1	1	3	4	4	6	7	7	9	9	10	11	13	17
	10	0	0	0	0	1	2	2	2	3	3	4	5	5	7
WHc	16	4	4	4	5	6	8	8	8	16	18	18	18	18	18
	17	0	1	1	3	5	6	7	8	10	13	13	15	16	18
	18	0	4	5	5	5	8	8	8	8	10	10	10	14	16
	19	1	2	3	5	7	8	9	11	13	15	18	18	18	18
	20	1	4	4	4	5	6	7	8	9	10	12	13	15	16
WHc Game 3	16	4	5	5	5	6	8	9	9	10	16	16	17	17	17
	17	0	2	7	7	8	10	10	10	11	11	12	18	18	18
	18	0	4	5	5	7	7	7	8	8	9	10	14	15	17
	19	1	1	2	3	3	3	4	6	14	14	18	18	18	18
	20	0	1	3	3	4	4	5	7	11	11	12	15	16	18

N=70 for each treatment. N=14 for each group.

Table A4.1. Distribution of individual C-rate in Game 2 (Rounds 6~23)

# of C	0~2	3~6	7~11	12~15	16~18
RM	39	17	8	3	3
WH	32	22	9	2	7
WHc	7	19	21	9	14
WHc Game 3	8	18	22	7	15

Note: Using the $\chi 2$-test (goodness-of-fit test), the distribution for the # of C can be separated into two significantly different groups: {RM, WH} and {WHc, WHc(3)}.

Table A4.2. Descriptive Statistics of individual C-rates in Game 2 (Rounds 6~23)

	RM	WH	WHc	WHc (Game 3)
Mean	3.629	4.914	8.9	9.071
Std.	4.553	5.410	5.572	5.668
Q1	0	1	5	4.25
Median	2	3	7	8
Q3	5	6.75	14	14

Note: The mean is obviously largest for WHc. From the difference of median and mean, RM & WH are slightly skewed to the right (mean > median), and WHc is also skewed to the right (mean > median). Also, the inter-quartile (the difference of Q3 and Q1) is much larger for WHc, while it is similar for RM and WH.

Table A5. Group level distribution of CC, CD, and DD

**Treatment**	**Group**	**p2(c)**	**CC**	**CD**	**DD**	**p-value (χ^2-test)**
	**1**	0.214	0.0159	0.3968	0.5873	0.0180
	**2**	0.258	0.1032	0.3095	0.5873	0.0098
**RM**	**3**	0.139	0.0238	0.2302	0.7460	0.8364
	**4**	0.179	0.0397	0.2778	0.6825	0.7009
	**5**	0.218	0.0476	0.3413	0.6111	0.9999
	**avg**	**0.202**	**0.0460**	**0.3111**	**0.6429**	**0.9942**
	**std**	**0.045**	**0.0343**	**0.0631**	**0.0696**	
	**6**	0.409	0.3333	0.1508	0.5159	<0.0001
	**7**	0.083	0.0079	0.1508	0.8413	0.9798
**WH**	**8**	0.333	0.1587	0.3492	0.4921	0.0031
	**9**	0.405	0.2381	0.3333	0.4286	<0.0001
	**10**	0.135	0.0238	0.2222	0.7540	0.7483
	**avg**	**0.273**	**0.1524**	**0.2413**	**0.6063**	**<0.0001**
	**std**	**0.154**	**0.1392**	**0.0960**	**0.1802**	
	**16**	0.615	0.4841	0.2698	0.2460	<0.0001
	**17**	0.488	0.3532	0.2738	0.3730	<0.0001
**WHc**	**18**	0.377	0.1627	0.4643	0.3730	0.6329
	**19**	0.571	0.3889	0.3810	0.2302	0.0024
	**20**	0.421	0.2063	0.4444	0.3492	0.3154
	**avg**	**0.494**	**0.3190**	**0.3667**	**0.3142**	**<0.0001**
	**std**	**0.100**	**0.1327**	**0.0919**	**0.0705**	
	**16**	0.571	0.3968	0.3651	0.2381	0.0004
	**17**	0.550	0.3929	0.3452	0.2619	<0.0001
**WHc**	**18**	0.461	0.2222	0.5000	0.2778	0.8798
**(Game 3)**	**19**	0.496	0.3929	0.2024	0.4048	<0.0001
	**20**	0.432	0.2738	0.3373	0.3889	<0.0001
	**avg**	**0.502**	**0.3357**	**0.3500**	**0.3143**	**<0.0001**
	**std**	**0.059**	**0.0821**	**0.1057**	**0.0769**	

Note: Hypothetical distributions over CC, CD, DD are calculated as p2(c)* p2(c), 2* p2(c) *[1- p2(c)], and [1- p2(c)]*[1- p2(c)] respectively. $\chi 2$-test (goodness-of-fit test) numbers for groups are included for the sake of completeness. And, observed CD % > hypo. CD % occurs only in Groups 1 & 5 (RM) and Group 23 (WHc-Game 3).

Note: We separate the matching into CD vs. non-CD (i.e., CC & DD) and use $\chi 2$ test to check if they fit random matching. The p-values for RM, WH, and WHc are .5636, .0000, and .0000, where p-value < .05 indicates non-random matching.

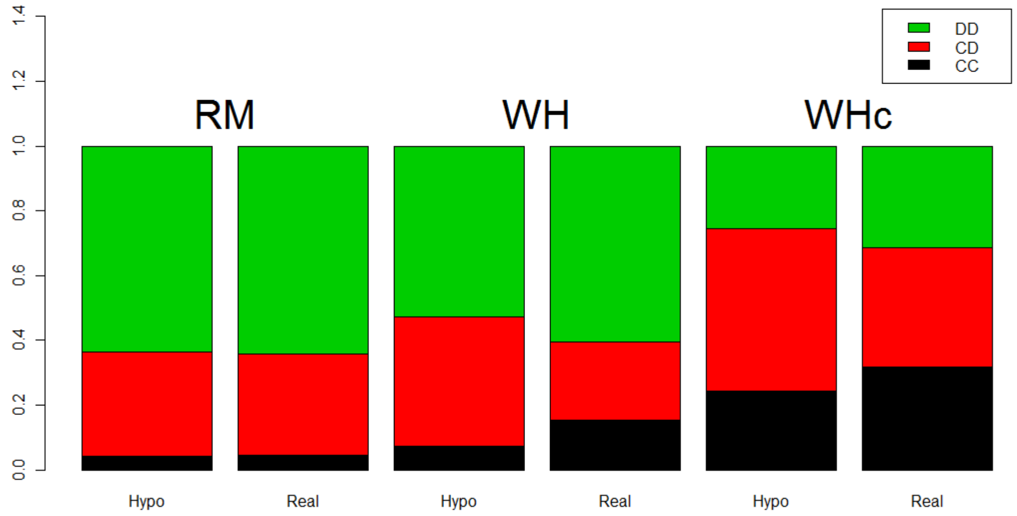

Figure A3. *Distributions of CC, CD, & DD (Game 2, Rounds 6~23): Hypothetical vs. realized. WH and WHc display significant AM effect.*

Table A6.1. Correlation coefficients of P(C) in Game 2 Rounds 1~5 vs. 6~23

Correlation N=70	RM	WH	WHc	WHc (Game 3)
Game 1 vs. Game 2 (1~5)	0.6849	0.4704	0.5896	0.5176
Game 1 vs. Game 2 (6~23)	0.5359	0.2919	0.4972	0.4397
Game 2 (1~5) vs. (6~23)	0.5604	0.6643	0.7220	0.7988

Note: Except for RM, it seems that Game 2 (rounds 1~5) has a stronger correlation than Game 1, with Game 2 (rounds 6~23). Both Game 1 and Game 2 (rounds 1~5) can be used as explanatory variable in P(C) in Game 2 (rounds 6~23). It seems that Game 2 (rounds 1~5) would have higher impact in WH treatments.

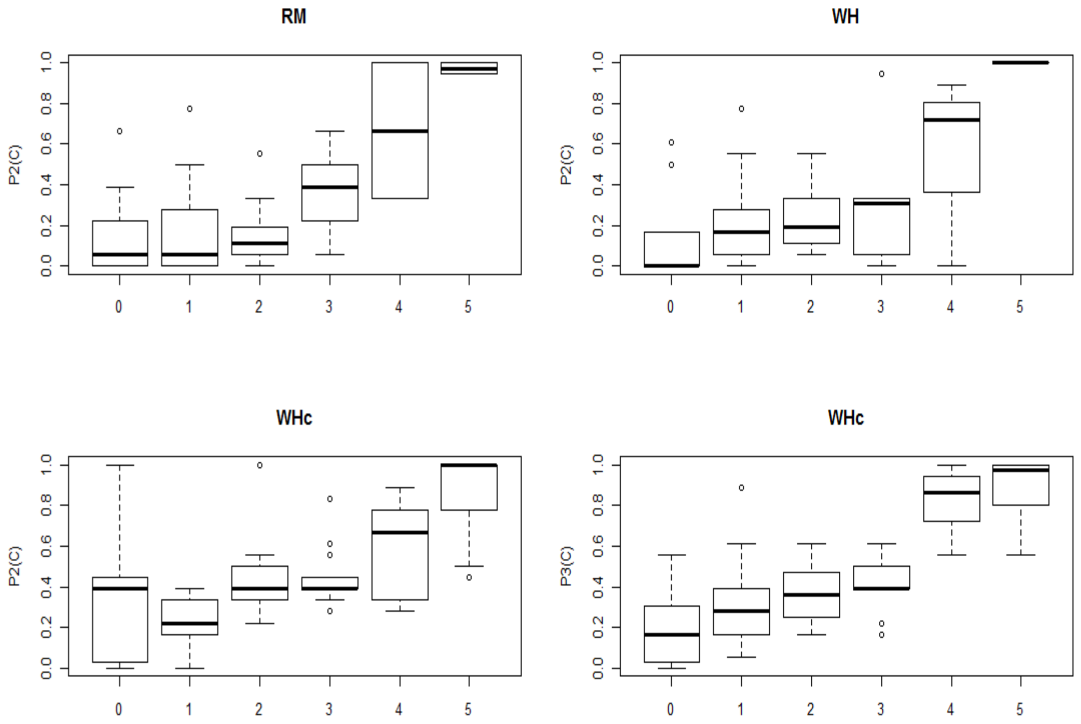

Figure A4. Probability of cooperation in Game 2 Rounds 1~5 vs. Rounds 6~23

Table A6.2. Distribution of #C individuals in Game 1

# of C	0	1	2	3	4	5
RM	10	11	25	8	9	1
WH	9	22	22	9	4	4
WHc	15	12	18	14	2	9

We observe in Table 6.2 that in both of #C=4 and =5 groups some treatments have too small sizes of sample. Merging the two classes yields numbers between 8 and 11 that matches the smallest in all other classes.

χ^2-tests that this distribution is not significantly different also on the individual level with N=70 for each sample, p = .2704, as additional evidence for non-bias in sampling.

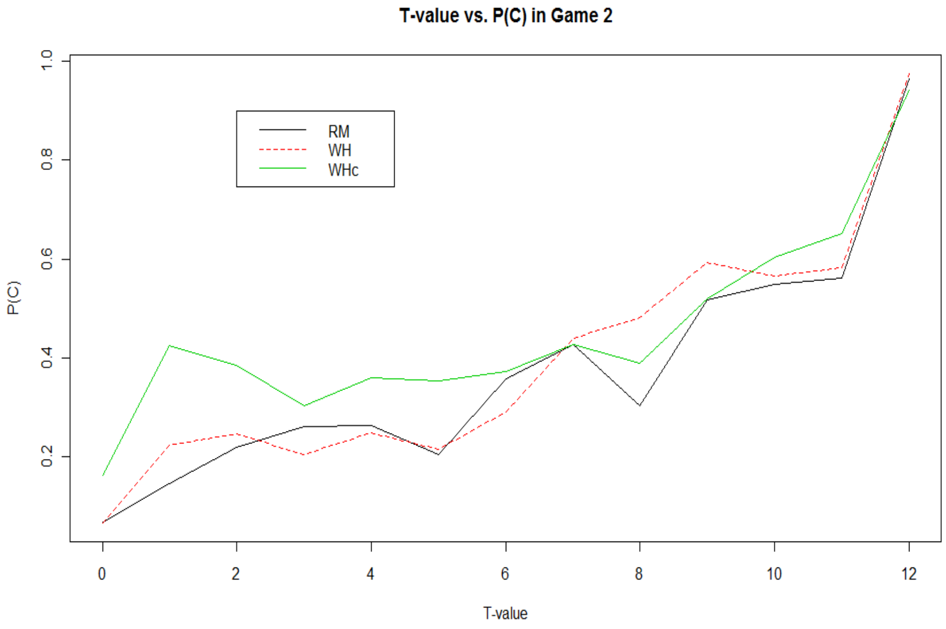

Figure A5. The probability of cooperation vs. matching score T in Game 2

General Notes on Logistic Regression

Logistic regression is a regression method used to deal with the case where the response (say, variable y) is dichotomous. Since the response is either 0 or 1, the logistic regression is to model the probability of y = 1 as

$$P(y=1 \mid x_1,\ldots,x_k) = \exp(\beta_0 + \beta_1 x_1 + \cdots + \beta_k x_k) \qquad (1)$$

where $x_1,\ldots,x_k$ are independent variables. The logistic regression is usually conducted via logarithm of *odds*, or

$$\log(odds) = \beta_0 + \beta_1 x_1 + \cdots + \beta_k x_k \qquad (2)$$

where the odds is defined as $P(y=1 \mid x_1,\ldots,x_k)/P(y=0 \mid x_1,\ldots,x_k)$.

The significance of independent variable x_i can be judged by odds ratio, or $\exp(\beta_i)$. However, like in the linear regression, the model with the largest R^2 is not necessarily the best model and there are no unique measures to determine the best model in logistic regression. Still, there are some measures which can be used to choose feasible models in logistic regression. We choose two frequently measures: **logarithm of likelihood** and **concordance**, and larger values of these two measures indicate a better fit. We can also use the logarithm of likelihood and the **number of parameters**, to avoid the possibility of **over-fit** (i.e., using too many independent variables). In addition, we shall check if the model assumption of logistic regression is violated. A

model with larger logarithm of likelihood and concordance, provided that the model assumption is not violated, is preferred.

Note that the model assumption is usually evaluated by the **goodness-of-fit** test. Two frequently used goodness-of-fit are Pearson and Deviance tests. However, as the number of groups increases, these two tests are more likely to falsely reject the null hypothesis. Another (Pearson-like) goodness-of-fit test, proposed by Hosmer and Lemeshow (1980), is used more often in practice, as in this paper. Note that the Hosmer-Lemeshow (H-L) test is to group residuals based on the values of the estimated probabilities. The default number of groups in H-L test is 10, as in this paper.

Regressions for the paper

Forms for the models considered are as follows, where *I* denotes indicator function.

Game 1: $\log it(p(C)) = \beta_0 + \beta_1 T + \beta_2 T^2 + \beta_3 T^3 + \alpha_1 \times \#C\, in\, Game1$

Table A7. Statistical performance of Game 1 model separate for each treatment

Treatment	log Likelihood	Hosmer Lemeshow	Concordance	# of Parameters
RM	-477.37	.583	78.8%	5
WH	-512.35	.143	82.2%	5
WHc	-690.40	.432	76.2%	4

We further compare the parameters for different treatments, as shown in Table A7.1. The values of parameters $\beta_0, \beta_1, \beta_2, \beta_3$ are similar for RM and WH, and the major difference is on α_1. It seems that the players for RM with higher cooperation level in Game 1 would have higher cooperation level in Game 2, comparing to those for WH. The players for WHc behave quite differently. (WHc has clearly higher β_0, which means lower initial inclination to play D, compared to RM and WH.)

Table A7.1. Estimated coefficients in Game 1 model

Treatment	β_0	β_1	β_2	β_3	α_1
RM	-3.0536	.8216	-.1536	.0097	.3006
WH	-2.6416	.8397	-.1603	.0106	.1142
WHc	-1.4998	.5324	-.1271	.0090	.1793

Note, we initially did a regression for all observations together with dummies for the treatments among others. But the Hosmer-Lemeshow goodness-of-fit test yields unsatisfactory results, i.e. $p < .05$, in all such models. Thus, we concluded that structurally the treatments are different and performed separate regressions for each as

shown above. In fact, we also experimented with other (treatment-separate) models, as follows. As it turns out, 'Game 1' model is the only one robust in all treatments. It also presents the best fit in RM and WH, is only slightly outperformed by the model 'Game 2 (#C=1,4,5)' in WHC.

Isolation: $\log it(p(C)) = \beta_0 + \beta_1 T + \beta_2 T^2 + \beta_3 T^3$

Individual: $\log it(p(C)) = \beta_0 + \beta_1 T + \beta_2 T^2 + \beta_3 T^3 + \sum_k \gamma_k \times I\{Player\ k\}$

Game 2 (1~5): $\log it(p(C)) = \beta_0 + \beta_1 T + \beta_2 T^2 + \beta_3 T^3 + \alpha_2 \times \#C\ in\ Game 2(1-5)$

Game 2 (#C=1,4,5):

$$\log it(p(C)) = \beta_0 + \beta_1 T + \beta_2 T^2 + \beta_3 T^3 + \alpha_2^* \times I\{\#C = 1,4,5\ in\ Game 2(1-5)\}$$

Table A7.2 Logistic Regression for P(C) in Game 2

		log Likelihood	Hosmer - Lemeshow	Concordance
RM	Individual	-462.69	.516	76.2%
	Isolation	-486.58	.310	72.6%
	Game 1	-477.37	.583	78.8%
	Game 2 (1~5)	-483.57	.851	77.3%
WH	Individual	-497.06	**.029**	82.2%
	Isolation	-513.94	**.011**	78.7%
	Game 1	-512.35	.143	82.2%
	Game 2 (1~5)	-509.01	.119	81.8%
WHc	Individual	-674.16	**.000**	74.5%
	Isolation	-687.40	.052	71.5%
	Game 1	-690.40	.432	76.2%
	Game 2 (1~5)	-670.86	**.030**	78.3%
	Game 2 (#C=1,4,5)	-664.55	.229	78.0%

Note: Shaded rows indicate best fitting models in respective treatments. Boldface indicates failed goodness-of-fit tests. The model "Game 1" is the best for RM and WH. "Game 2 (t=1-5)" would be best for WHc, if not for its HL-test failure. Yet, replacing it with the dummy, "Game 2 (#C=1,4,5)" gets it done.
